# Impairment of Long-Term Plasticity of Cerebellar Purkinje Cells Eliminates the Effect of Anodal Direct Current Stimulation on Vestibulo-Ocular Reflex Habituation

**DOI:** 10.3389/fnins.2017.00444

**Published:** 2017-08-03

**Authors:** Suman Das, Marcella Spoor, Tafadzwa M. Sibindi, Peter Holland, Martijn Schonewille, Chris I. De Zeeuw, Maarten A. Frens, Opher Donchin

**Affiliations:** ^1^Department of Biomedical Engineering and Zlotowski Center for Neuroscience, Ben Gurion University of the Negev Be'er Sheva, Israel; ^2^Department of Neuroscience, Erasmus Medical Center Rotterdam, Netherlands; ^3^Department of Integrative Neurophysiology, Center for Neurogenomics and Cognitive Research, Vrije Universiteit Amsterdam Amsterdam, Netherlands; ^4^Netherlands Institute for Neuroscience Amsterdam, Netherlands; ^5^Faculty of Social and Behavioral Sciences, Erasmus University College, Erasmus University Rotterdam, Netherlands

**Keywords:** tDCS, LTP, purkinje cell, vor, cerebellum

## Abstract

Anodal direct current stimulation (DCS) of the cerebellum facilitates adaptation tasks, but the mechanism underlying this effect is poorly understood. We have evaluated whether the effects of DCS effects depend on plasticity of cerebellar Purkinje cells (PCs). Here, we have successfully developed a mouse model of cerebellar DCS, allowing us to present the first demonstration of cerebellar DCS driven behavioral changes in rodents. We have utilized a simple gain down vestibulo-ocular reflex (VOR) adaptation paradigm, that stabilizes a visual image on the retina during brief head movements, as behavioral tool. Our results provide evidence that anodal stimulation has an acute post-stimulation effect on baseline gain reduction of VOR (VOR gain in sham, anodal and cathodal groups are 0.75 ± 0.12, 0.68 ± 0.1, and 0.78 ± 0.05, respectively). Moreover, this anodal induced decrease in VOR gain is directly dependent on the PP2B medicated synaptic long-term potentiation (LTP) and intrinsic plasticity pathways of PCs.

## Introduction

Transcranial direct current stimulation (tDCS) modulates cerebellar dependent motor learning tasks (Jayaram et al., 2012; Hardwick and Celnik, 2014; Herzfeld et al., 2014; Avila et al., 2015) by applying a weak constant electrical current (amplitude <2 mA) through scalp electrodes. This technique allows us to stimulate the target region by the positive (anodal) or negative (cathodal) current (Das et al., 2016). Data collected in humans suggests that polarity specific effects of tDCS may be obtained by changing cerebellar cortical excitability (Galea et al., 2009). However, the mechanism behind tDCS dependent modulation of motor learning is unclear (Das et al., 2016). To optimally use tDCS in various cerebellar dependent motor learning disorders, a better understanding of mechanisms is vital (Ivry and Spencer, 2004; Xu-Wilson et al., 2009; Bastian, 2011; Hardwick and Celnik, 2014; Benussi et al., 2015).

Various animal models of DCS (direct current stimulation that is not transcranial) serve in exploration of the mechanism of tDCS (Creutzfeldt et al., 1962; Bindman et al., 1964; Purpura and Mcmurtry, 1965). In these models, a small part of the skull is removed at the site of stimulation in order to reduce the inter-subject variability of transcranial-conductance.

Our current study aims to explore the mechanism of action of DCS on cerebellar learning. To probe polarity specific effects of DCS on cerebellar learning, we employed a gain-down vestibulo-ocular reflex (VOR) adaptation task. The VOR aims to compensate for head movement by making an eye movement in the opposite direction, in order to stabilize the image on the retina (Probst et al., 1986). This compensatory eye movement can be adapted based on mismatched visual input, a process that requires the cerebellum (Kawato and Gomi, 1992). Here we presented a sinusoidal optokinetic stimulation by using a 360° virtual environment and vestibular stimulus by using a turntable in phase, resulting in a decrease of the response to the same vestibular stimulus in the dark (Tempia et al., 1991). The turntable mimics the head movement while the movement direction of the virtual environment demands orientation specific compensation of the eye movement (similar to the natural environment).

The gain-down adaptation of the VOR (Tiliket et al., 1993) may depend partly on both the cerebellar flocculus and the downstream vestibular nuclei (VN) (Lisberger and Fuchs, 1974; Ito, 1982). To test the importance of Purkinje cell (PC) plasticity in polarity-specific DCS modulation, we investigated L7-PP2B mice, lacking postsynaptic and intrinsic plasticity of PC (Schonewille et al., 2010). Our prediction is that at least some DCS effects (caused either by anodal or cathodal stimulation) would be compromised in this mutant because DCS has an extensive modulatory role on PC dendrites (Chan and Nicholson, 1986; Chan et al., 1988).

A rodent model of DCS has been validated in cortical spreading depression (Liebetanz et al., 2006a) and epilepsy (Liebetanz et al., 2006b). Anodal stimulation of frontal cortex enhances the Blood-oxygen-level dependent (BOLD) signal, an indication of higher neuronal activity (Takano et al., 2011). Furthermore, DCS alters neocortical plasticity not only by altering pre-synaptic sensitivity (Márquez-Ruiz et al., 2012) but also by promoting brain-derived neurotrophic factor (BDNF) dependent long-term potentiation (LTP) (Fritsch et al., 2010). As the plasticity mechanisms of the cerebellar cortex are different from those in neocortex (Hansel, 2005; Lamont and Weber, 2012) there is ample justification for an animal model of cerebellar DCS. Moreover, the cerebellum is ideal to identify the mechanism(s) of DCS because—(i) the structure of rodent cerebellum is clear and accessible, (ii) the plasticity mechanisms are well studied, and (iii) there is a wide range of mutant mouse models available to test which pathways are functionally relevant (De Zeeuw et al., 2011). Therefore, the present study focuses not only on developing an animal model of cerebellar DCS but also utilizes one of the most important mutant mouse models to unravel the role of PC plasticity in mediating DCS effects on VOR adaptation.

## Methods

### Summary of methodology

C57BL/6 (wild type, *N* = 24) and L7-PP2B (LTP deficient mutants, *N* = 22) mice were implanted with a DCS-implant for administration of DCS over the cerebellum. DCS was applied to separate groups of mice as anodal, cathodal or sham-stimulation. Eye movements were recorded using an infrared-sensitive CCD camera during horizontal VOR gain-down adaptation learning. In testing sessions, the eye response to vestibular stimulation, i.e., the motion of the table, (amplitude of 5° at 1 Hz frequency) in the dark was recorded. In training sessions, vestibular and visual stimulation (amplitude of 5° at 1 Hz frequency) were coupled so as to cause reduction of the VOR gain. Two baseline test sessions were followed by 10 min of DC stimulation and then by an additional baseline test session. There were then 5 training sessions of 5 min each, each followed by a test session. We subsequently compared the reduction of VOR gain in the different stimulation groups and across strains.

### Experimental paradigm

Mice were habituated to the experimental apparatus for a minimum of 2 days to reduce the novelty-induced anxiety and restrain-stress after they recovered from the surgery.

**Figure 1:**
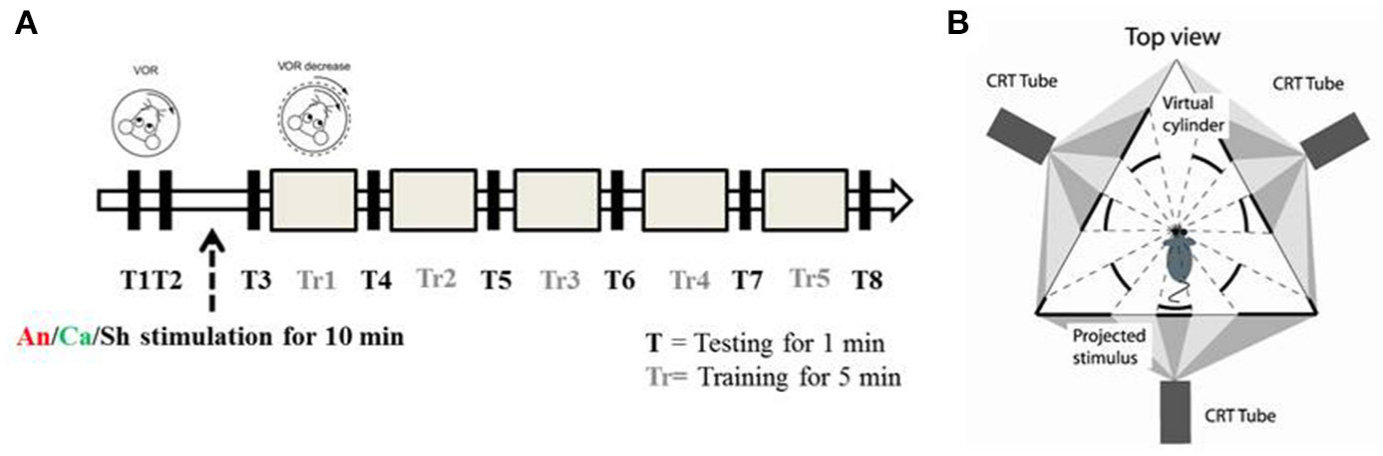
Experimental paradigm and set up **(A)** Schematic diagram of the experimental paradigm. *T* represents a testing session during which the animal is exposed to VOR in the dark by moving the turntable in a sinusoidal manner (5° amplitude at 1 Hz). *Tr* represents the training session during which the animal is presented with a sinusoidal visual cue which is in phase with the table movement. After two testing sessions (*T1* and *T2*) the animals were randomly assigned to the anodal (*An*), cathodal (*Ca*) or sham (*Sh*) stimulation group. **(B)** Schematic diagram of the experimental apparatus. Top down view describes the position of the mouse in relation to the virtual environment created by three projectors.

Each experiment consisted of 8 test (T) and 5 training (Tr) sessions. The duration of each test session was 1 min, and the duration of each training session was 5 min. In test sessions, a sinusoidal vestibular stimulation which was generated by moving the table with a 5° amplitude at 1 Hz frequency, was applied in the dark. Eye movements were recorded simultaneously. In training sessions, in phase vestibular and optokinetic sinusoidal stimuli (5° amplitude at 1 Hz frequency) were given (Figure 1A), in order to reduce the VOR gain. Eye movements were continuously recorded.

Every experiment was initiated by two baseline measurements of VOR (T1 and T2). Then the mice were randomly divided into 3 groups, and received anodal, cathodal or sham DC stimulation. The current amplitude was ramped up over 30 s to 113.2 μA and kept constant for 10 min (positive polarity for the anodal group, negative polarity for the cathodal group). For the sham group, amplitude was then immediately ramped down (over 30 s) while for the anodal and cathodal groups current was maintained for 10 min of stimulation. After the stimulation, another session of testing (T3) was conducted and then gain-down adaptation learning training was initiated. A testing session was conducted to calculate the learning rate after every training session (Figure 1A).

### Experimental procedure

#### Animals

C57BL/6 (*N* = 24) mice were acquired from Charles River laboratories, Inc. (Wilmington, MA, USA). L7-PP2B mutants (*N* = 22) were bred in Erasmus MC, Rotterdam. Mouse lines used in this study have been described previously (Schonewille et al., 2010). Three to four mice were caged together in temperature-regulated (22 ± 1°C) housing with a 12:12 light-day cycle. Behavioral experiments were performed in the light cycle. Food and water was provided *ad libitum*. All experiments were reviewed and approved by the Erasmus animal ethics committee and conducted in accordance with Animal Welfare Committee of the Erasmus University and the European Communities Council Directive (86/609/EEC).

#### Surgery

Mice, aged 10–12 weeks, were handled for 2 days before the surgery to reduce the effect of handling-induced stress. The surgical procedure was performed under sterile conditions. Isoflurane (5% induction, 1.5% in 0.5 L/min O_2_, and 0.2 L/min air) was administered as an anesthetic drug while body temperature was regulated around 36.5 ± 0.5° via a feedback-controlled heating pad. Breathing profile was continuously monitored. After shaving the head, a 1 cm long mid-sagittal incision was given. The bone was etched (37.5% phosphoric acid, Kerr, CA, USA) and a primer (Optibond, Kerr, CA, USA) was applied. To immobilize the animal during eye tracking, a pedestal containing two M1.4 nuts was glued to the skull using dental acrylic (Charisma, Flowline, Hereaus Kulzer GmBH, Germany).

**Figure 2:**
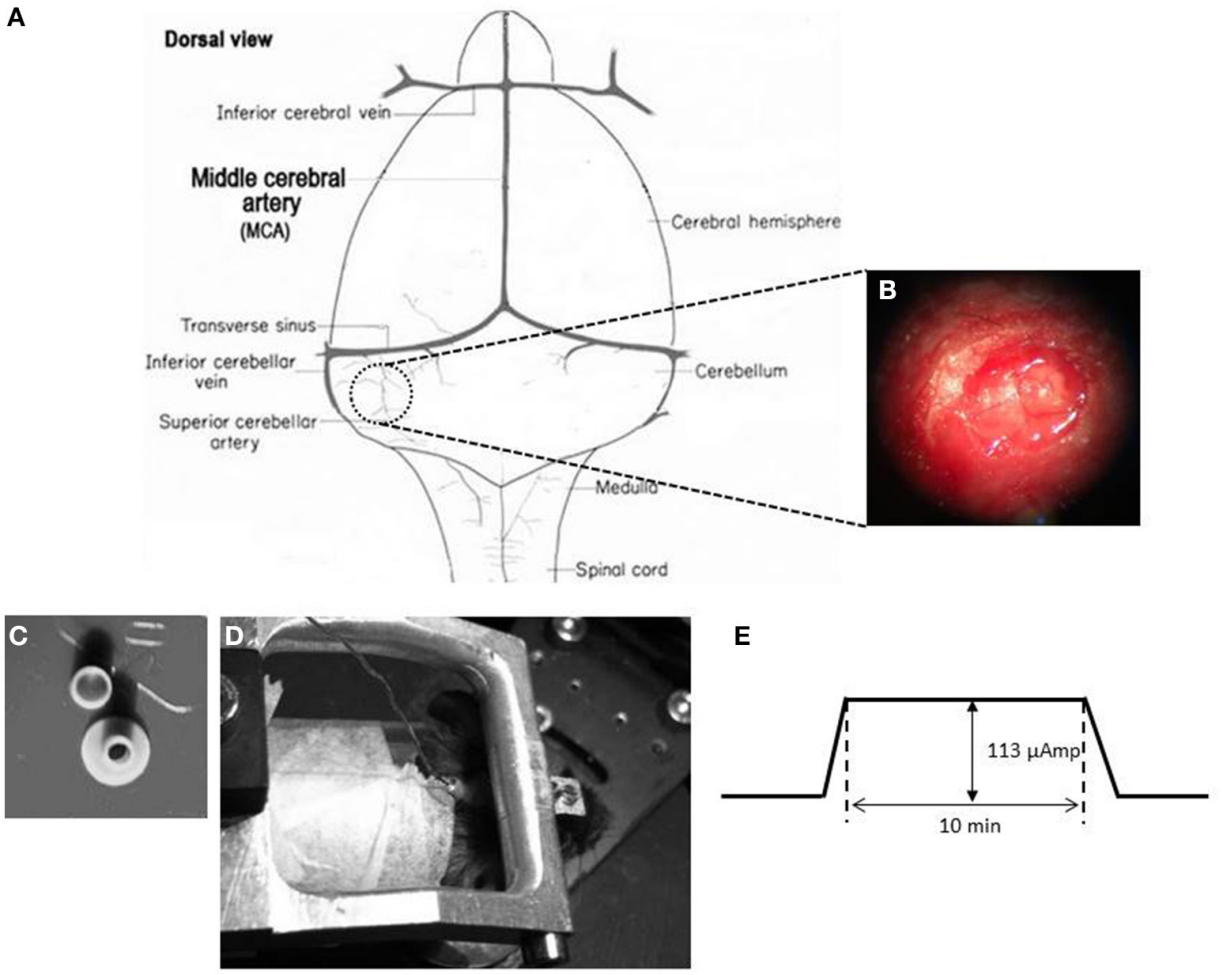
DCS location and procedure **(A)** Schematic representation of craniotomy for placement of implant over the cerebellum of a mouse brain. **(B)** Craniotomy. Anatomical location for the DCS-implant placement. **(C)** DCS implant. The DCS chamber serves as a bridge between the stimulating electrode and the brain. Above the chamber is the cap that serves to protect the brain from infection. **(D)** Stimulating the mouse cerebellum. The DCS chamber is filled with saline (0.9% NaCl) solution. A silver wire that touches the saline solution but not the dura directly is connected to the current generator (SUI-91, Isolated current source). During stimulation the mouse is awake but head restrained. **(E)** Stimulation paradigm. DCS is ramped up to 113 μAmp. The current is maintained at its peak value for 10 min. After the stimulation the current is ramped down.

In order to place a DCS implant, a circular craniectomy (approximately 2 mm in diameter) on the left occipital bone was performed after careful removal of the neck-muscles (vertical and horizontal) (Figures 2A,B). The placement was on the center of the left parietal bone (by keeping the superior cerebellar artery at the center of the implant). A lubricating ointment (Duratears, Alcon Nederland BV, NL) was applied epidurally to protect the exposed area of brain from drying. The DCS implant (Figure 2C) was placed identically in all animals using an anatomical marker (Figures 2A,B) and then glued to the skull using cyanoacrylate gel (Plastic One Inc., VA, USA).

The mice were given an analgesic (0.1 ml/mg of body weight Buprenorphine/Temgesic) and placed under an infrared heating lamp until the animals started to move. Mice were allowed at least 4–5 days to recover before recordings were performed.

### Apparatus

#### Visual and vestibular stimulation

Mice were head-fixed in a restrainer, which was fixed onto the center of a turntable, placed at the center of an isolateral triangle made by three projector-screens. A panoramic virtual reality display with 360° field of view was created by projecting monochrome green dots on to those screens (Figure 1B). Horizontal rotation of the turntable was driven by a servomotor (Mavilor-DC motor 80, Infranor, Spain). Visual stimuli and movement of the turntable were under control of in-house software written in C++. Training and testing sessions were evoked by rotating the dots and/or the turntable sinusoidally. During each session, stimuli were ramped up to their peak velocity in 5 s for a smooth transition from static to dynamic state. They were also ramped down at the end.

#### Eye movement recordings

Eye movements were recorded with an infrared video system (ETL-200 with marker tracking modifications; ISCAN, Burlington, MA). The camera and lens were mounted under the table surface to reduce hindrance of the mouse vision. A hot mirror which was transparent to visible light and reflective to infrared light was used. The eye was illuminated with three infrared LEDs. The camera, mirror and LEDs were all mounted on an arm that could rotate about the vertical axis over a range of 26.12° (peak to peak). Eye movement recordings and calibration procedures were similar to those described by Stahl et al. (2000). Images of the eye were captured at 120 Hz with an infrared-sensitive CCD camera. The eye image contained a bright corneal reflection and a dark pupil reflection. The image was focused by manipulating the offset and the gain of the detectors through the ISCAN software. From this image, x and y positions of each of the three markers were recorded in real time giving their location on a 512 × 256-pixel grid, with a resolution of one-third pixel horizontally and one-tenth pixel vertically (van Alphen et al., 2010). These x and y translational positions of eye on the grid were converted into the angular rotation of the eyeball by the ISCAN system (resolution of 0.2° over a ±25° horizontal and ±20° vertical range using the pupil/corneal reflection difference). The horizontal and vertical pupil position data from the ISCAN were output as ±5 VDC signals. A delay of 30 ms in the eye movement signal was introduced by the video system. Furthermore, this output signal was low-pass filtered with a cutoff frequency of 300 Hz (Cyberamp 380; Axon Instruments, CA, USA), sampled at 1 kHz and stored for offline analysis.

#### Direct current stimulation

A low amplitude (113 μA) of continuous DCS was applied using a constant current stimulator (SUI-91, Isolated current source, Cygnus Technology Inc., NC, USA; range = 0.1 μA—10 mA). This intensity corresponded to a current density of 3.6 mA/cm^2^ (Liebetanz et al., 2009). Currents were applied to the epidural surface of the cerebellar cortex through a circular DCS implant with a defined contact area (2 mm inner diameter). Prior to stimulation, the electrode was filled with saline solution (0.9% NaCl). A silver wire electrode connected to the stimulation device was attached to the DCS implant such that the tip of the silver wire touched the top level of the saline solution but did not touch the brain directly. This circular active electrode (Figure 2C) was chosen to create a symmetric current density without any edge effects (Ambrus et al., 2011). A disposable foam electrode (Kendall Medi-Trace mini resting ECG electrode, Davis medical products Inc., CA, USA), was placed onto the ventral thorax of the animal to complete the circuit. The entire circuit was connected through a multimeter to check online current amplitude. Mice were awake during DCS to prevent possible interactions between DCS effects and anesthetic drugs. In addition, mice were introduced to the adaptation task right after the stimulation to quantify acute effects of stimulation. To avoid stimulation break effects (Liebetanz et al., 2009), the current intensity was ramped up and down gradually over 30 s.

### Data analysis

Custom routines written in MATLAB (The MathWorks Inc., Natick, MA, USA) were designed and employed for automated offline data analysis. The position signal was shifted 30 ms back in time to correct for the camera delay. A median filter (width 50 ms) with a low-pass cutoff of 10 Hz was applied to smooth the position data before transforming to velocity domain by a Savitski-Golay differentiating filter (frequency 50 Hz with a 3° polynomial). Rapid eye movements were detected and removed via a velocity threshold (150°/s). Then a 3 Hz FIR Butterworth low pass filter of 50 ms width was applied.

The processed data was divided into non-overlapping epochs of 2 s (corresponding to two cycles of the stimulus). Amplitude data was obtained by fitting sine waves to the eye movement data in custom-made Matlab curve fitting routines using the least-squares method. Median amplitude values of the eye movement were calculated from the fitted sine waves. Gain was calculated for,

(1) $$\begin{array}{r} G_{T}=\frac{E_{Tn}}{S}\;\begin{array}{c} \text{E}=\text{fit eye amplitude of a testing session},\;n=1\\ \text{ through }8,\\ \text{S}=\text{stimulus amplitude}, \end{array} \end{array}$$

each testing session as the ratio between the fit eye velocity amplitude and stimulus velocity amplitude (***S***).

Mice were excluded when the absolute difference between baseline gains (*G*_*T*1_–*G*_*T*2_) was > 0.2. The baseline gain (***G***_*B*_) was set as the mean of gains in *G*_*T*1_ and *G*_*T*2_. Normalized gain (***G***_*N*_) was also calculated for every test session.

(2) $$\begin{array}{r} G_{N}=\frac{G_{B}-G}{G_{B}+G} \end{array}$$

### Statistical analysis

Statistical analysis of the data was performed using SPSS 20.0 (SPSS, Chicago, IL). A three way mixed-ANOVA with repeated measures was used to compare interaction and group effects, as the data showed a normal distribution. Significance levels were set to 0.05. Later on, a Bonferroni corrected *post-hoc* analysis was applied to find intra-/inter-group interactions. Values are represented here as mean ± SEM.

## Results

### Degree of adaptation at the end of training session

**Figure 3:**
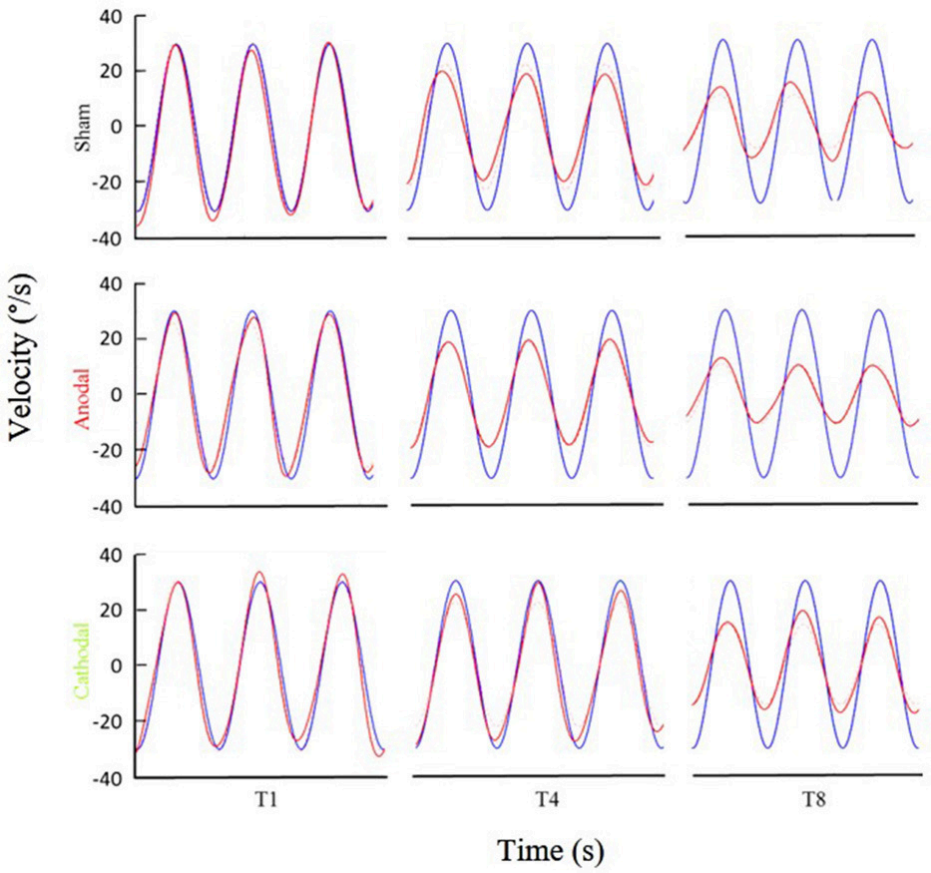
Examples of eye movement in different stimulation conditions. Examples of filtered eye velocity illustrate results from mice that exhibited a decrease in the VOR after training with sham (top panels), anodal (middle panels) and cathodal (bottom panels) stimulation. Blue is vestibular stimulus and red is eye amplitude (solid red line is filtered eye-velocity, dotted red line fitted sine wave). Eye-trace of each stimulus condition has been presented during pre-training (T1), after first-training (T4) and after final-training (T8) in the left, middle and right panels, respectively.

**Figure 4:**
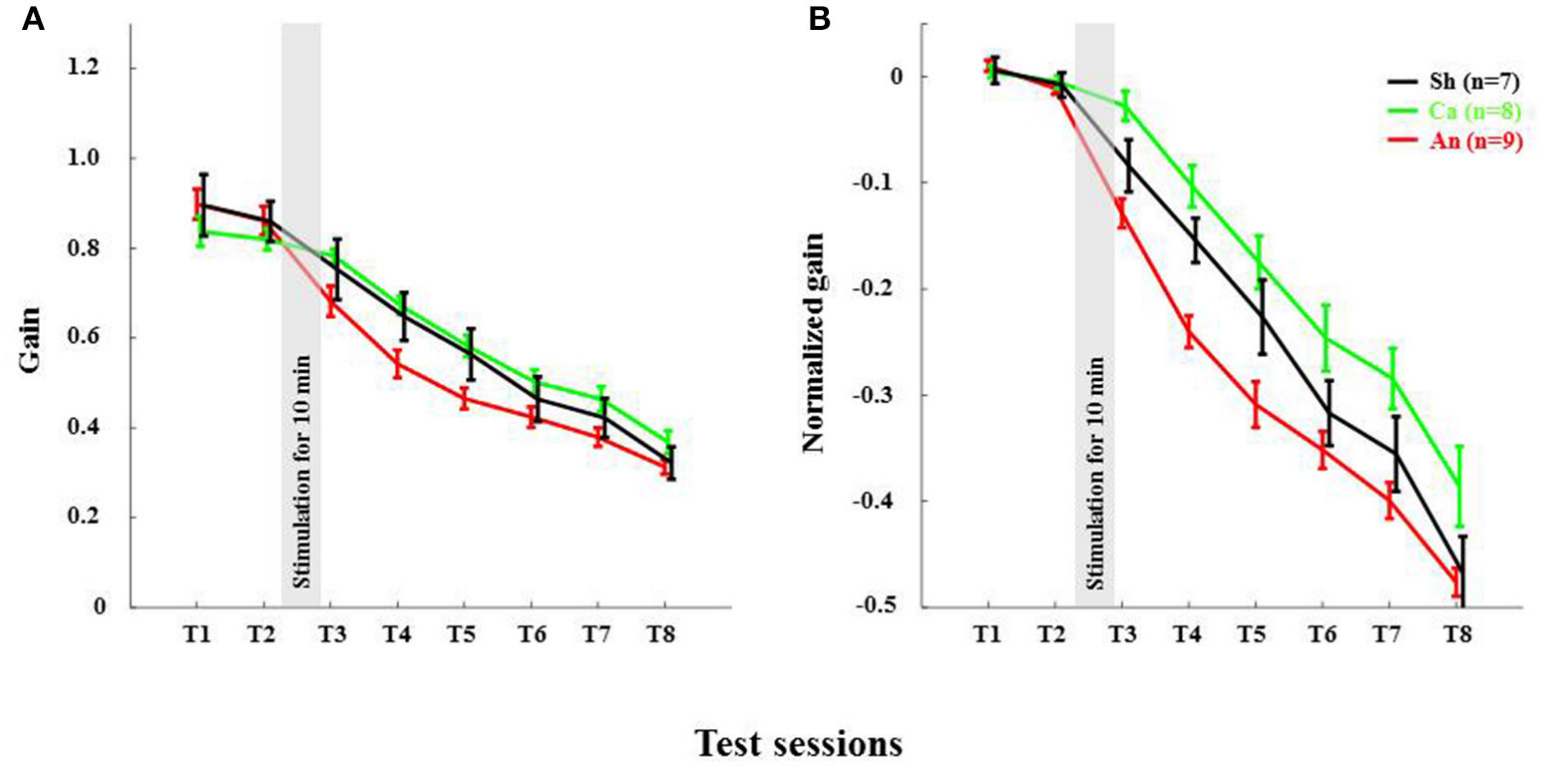
Anodal stimulation reduces VOR gain acutely in wild type C57BL/6 mice. **(A)** Time course of gain reduction due to adaptation: Changes in mean VOR gain during VOR-decrease training. The VOR was tested pre- and post-training by measuring the eye movement response to the vestibular stimulus. **(B)** Time course of normalized gain reduction due to adaptation: Trial-to-trial changes in mean normalized VOR gain during VOR-decrease training. Black, Green and Red lines are for sham, cathodal and anodal stimulation conditions, respectively. The gray bar indicates the stimulation period. Error bars represent SEM.

**Figure 5:**
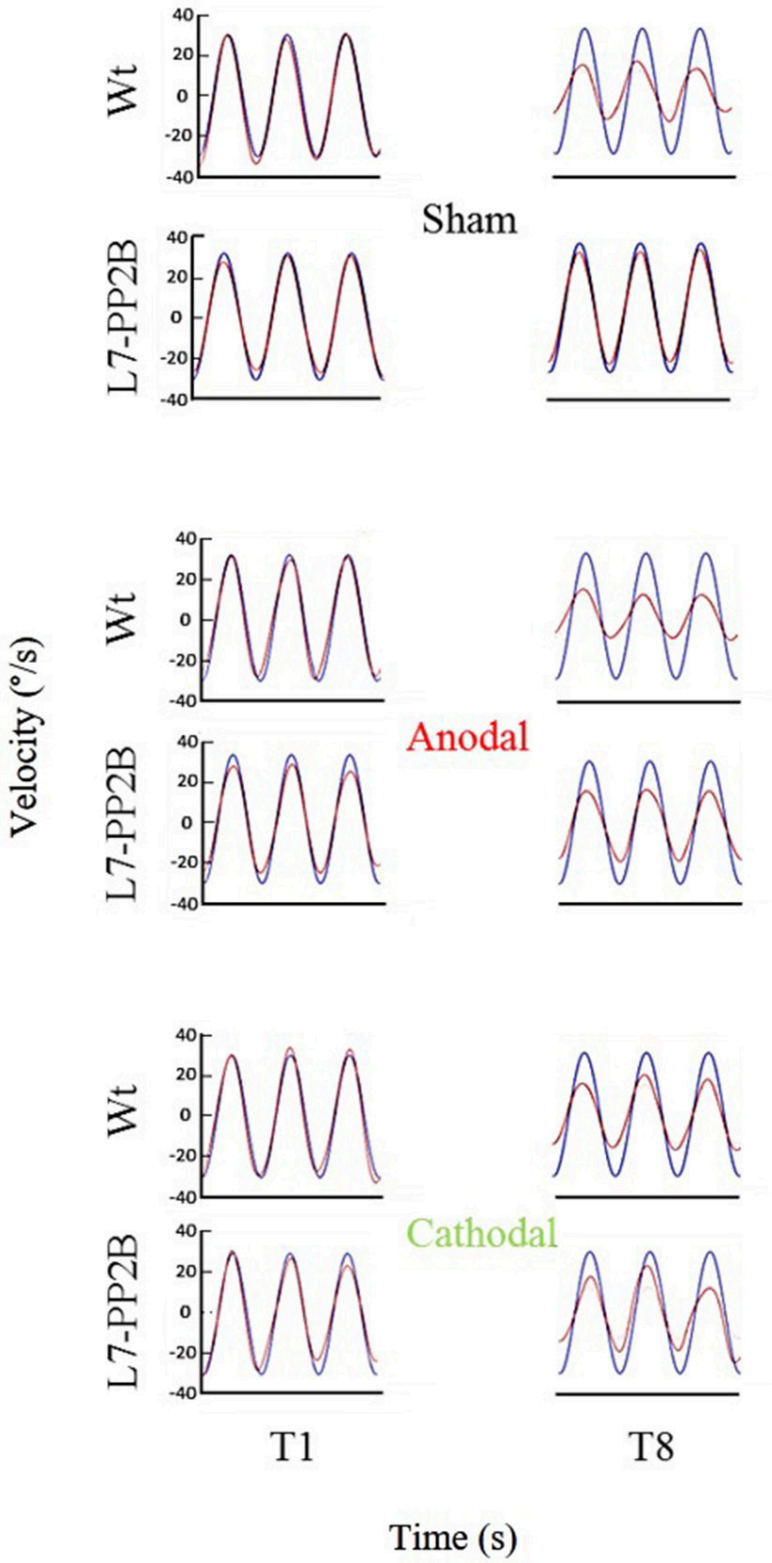
Eye amplitude show clear deficit in learning of L7-PP2B mice in all three stimulus conditions. Example filtered eye velocity traces illustrate typical results from mice of both genetic backgrounds before (T1) and after (T8) adaptation. Blue is vestibular stimulus and red is eye velocity (solid red line is filtered eye-signal, dotted red line is the fitted sine wave). Eye amplitude decreases from pre- to post-training sessions (T1 and T8, respectively) in wild type mice. In contrast, L7-PP2B undergoes little change between T1 and T8 sessions.

**Figure 6:**
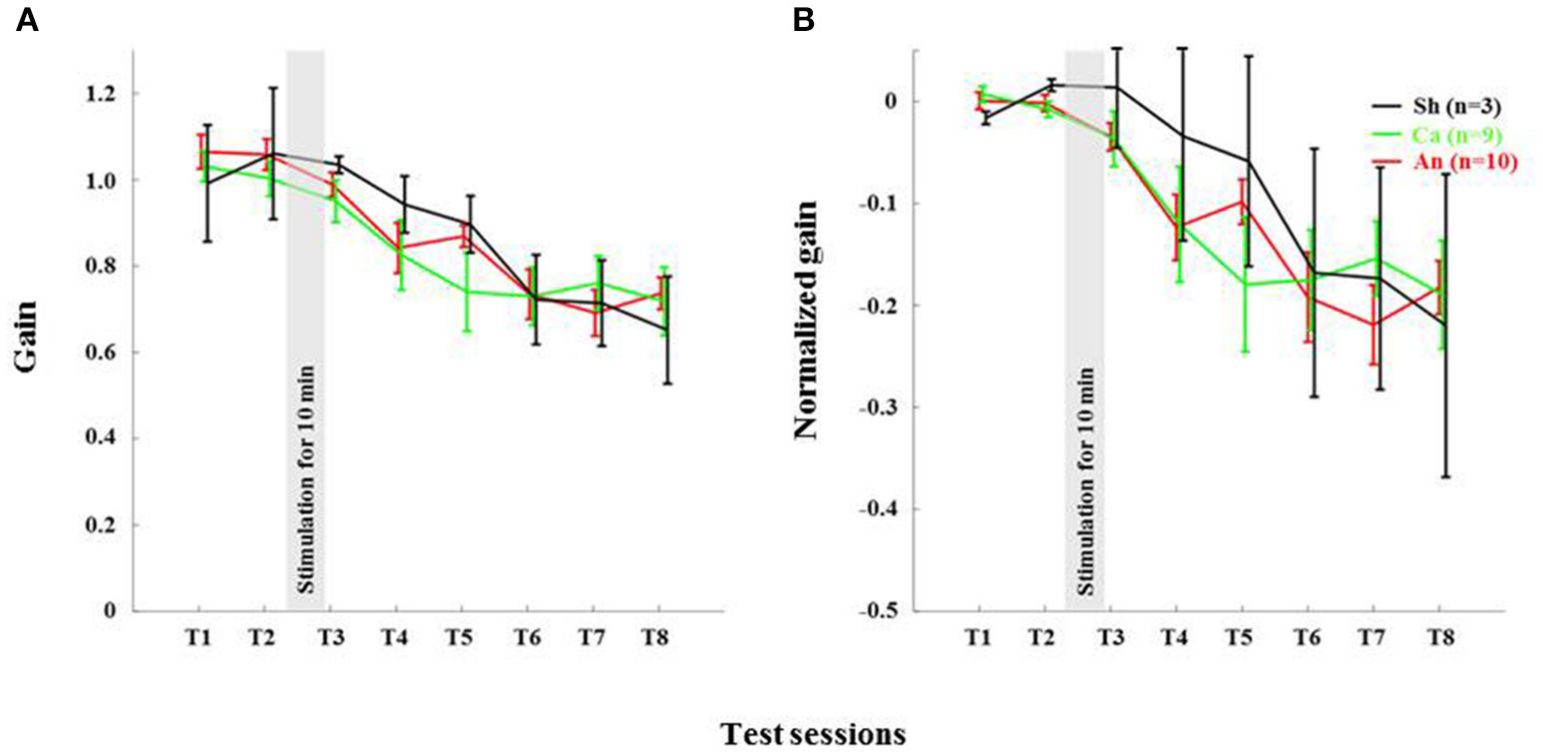
Genetic ablation of PC plasticity in L7-PP2B mice abolishes effects of anodal stimulation on gain-down adaptation. **(A)** Plot of gain during gain-down adaptation: Changes in VOR gain during VOR-decrease training in L7-PP2B mice. The VOR was tested pre- and post-training by measuring the eye movement response to the vestibular stimulus. **(B)** Plot of normalized gain throughout the course of the behavioral paradigm: Changes in VOR gain during VOR-decrease training. Black, Green and Red lines are for sham, cathodal and anodal stimulation conditions, respectively. The gray bar indicates the stimulation period. Error bars represent SEM, because of large SEM we do not find any significant difference between the groups.

The VOR gain-down adaptation paradigm caused a gradual reduction in VOR-amplitude in all mice (Figures 3–**6**). Initially, the amplitude of the eye movement was similar to the stimulus amplitude; i.e., the gain at T1 for C57BL/6 and L7-PP2B mice was 0.88 ± 0.03 and 1.03 ± 0.03, respectively (Figures 4, **6**). The baseline VOR gain in L7-PP2B of more than 1 indicated that the eye amplitude overshot the head amplitude in these mice. After being subjected for 25 min to the gain-down training, the amplitude of VOR at T8 was reduced for both C57BL/6 (raw T8 gain = 0.33 ± 0.03) and L7-PP2B (raw T8 gain = 0.70 ± 0.03) group. In our multivariate ANOVA on the non-normalized data, the main effect of training over the time course was highly significant, *F*_(7, 34)_ = 46.20, *p* < 0.001. However, comparison of the sham stimulation data showed that the degree of adaptation was significantly higher in C57BL/6 than L7-PP2B mice, *F*_(5, 40)_ = 14.94, *p* < 0.001.

### Reduction of gain in C57BL/6 and L7-PP2B mice

The reduction in gain made across the eight test sessions was strongly dependent upon the genetic composition of mice, *F*_(7, 38)_ = 4.98, *p* < 0.001. We sought to find out at which steps the gain was maximally reduced between C57BL/6 and L7-PP2B mice. To do that, we checked the gain difference between two successive test sessions and then compared that across the mouse types. The tests of within-subjects contrasts illustrated that the gain reduction from T5 to T6 [*F*_(5, 40)_ = 2.48, *p* < 0.05] and from T7 to T8 [*F*_(5, 40)_ = 2.66, *p* < 0.05] was significantly greater for C57BL mice compared to the L7-PP2B mice.

### Effects of DCS on VOR adaptation

ANOVA further indicated that DCS polarity had a significant modulatory role on the gain reduction, *F*_(14, 70)_ = 2.07, *p* < 0.05, suggesting that the amplitude of gain decrease across the eight tests (from T1 to T8) was dependent upon stimulus polarity. Moreover, the gain decrease across eight test sessions yielded a significant interaction between stimulus polarity and genetic background of the mice (C57BL/6 and L7-PP2B mice, [*F*_(7, 35)_ = 2.52, *p* < 0.05]. In the following sections, we discuss how the modulatory role of DCS was altered depending on the mouse type.

### Anodal stimulation reduced VOR gain in C57BL/6 acutely

The anodal stimulation triggered faster initial VOR gain reduction compared to the cathodal stimulation [*F*_(2, 21)_ = 9.56, *p* < 0.001, Figure 4A] in wild type mice. There was a significant post-stimulation reduction of gain at T3 (pre-training reduction of gain) in the anodal group compared to the cathodal group. The contrast analysis, T2 vs. T3, comparing the raw gain at T2 with that made in T3, was statistically significant [*F*_(2, 21)_ = 6.01, *p* < 0.01]. Interestingly, the anodal, sham and cathodal groups finished at the same degree of adaptation (T8), although the anodal group showed significant initial reduction in VOR gain.

Next, we normalized the gain of every mouse to its own baseline to provide a comparable measure of gain for all animals. Normalized gain (Figure 4B) depicted a clear polarity-dependent divergence. The initial post-stimulation period showed that the cathodal stimulation significantly decelerated gain reduction compared to the anodal stimulation. The reduction of gain in the sham condition—as expected—remained between the rate in the anodal and the cathodal conditions (Figure 4B).

### Deletion of PP2B in PC abolished anodal effect

Anodal stimulation lost its modulatory role when potentiation was eliminated from PCs (**Figures 6A,B**). Anodal stimulation failed to improve learning in L7-PP2B mice (T8 gain = 0.74 ± 0.04), compared to the sham group (T8 gain = 0.65 ± 0.08; Figure 5). Moreover, anodal stimulation could not reduce the baseline gain in these mutants (T2 gain = 1.06 ± 0.04, T3 gain = 0.99 ± 0.04). The large error bars in the sham condition is due to low sampling numbers (*N* = 3). Moreover, we think that chronic mutation (deletion of LTP in PCs) leads to the adoption of various adaptation mechanisms in the network. Therefore, when an external current stimulus was applied the network showed varied responses to cope with the situation. This could be the cause for finding a large variability in the stimulation groups.

An hour long sinusoidal oscillatory stimulus led to decrease in VOR gain (approximately to 28%) across various species (Tempia et al., 1991; Dow and Anastasio, 1998; Clément et al., 2002). The cause of this VOR gain reduction in rodents has been pointed out as habituation rather than learning (Tempia et al., 1991). Therefore, we think that the gain reduction (27 ± 2%) in L7-PP2B mice (similar to Schonewille et al., 2010) across all three stimulation-conditions is due to the habituation.

## Discussion

Our study demonstrates three major findings of the polarity specific effects of DCS on VOR gain-down adaptation. First, anodal stimulation of cerebellar cortex decreases VOR gain acutely compared to the cathodal stimulation condition in C57BL/6 control mice. Second, despite differences in initial post-stimulation reduction in gain amplitude, the final gain reduction is similar in the anodal and the cathodal stimulation groups of C57BL/6 control mice. Third, our data, remarkably, shows when potentiation of the PCs is genetically ablated in L7-PP2B mice, anodal stimulation no longer led to VOR gain reduction. Hence, our interpretation is that anodal stimulation driven VOR gain reduction depends on a PP2B-dependent PC potentiation pathway, either at the upstream dendritic level or at the downstream axonal level where PCs innervate VN neurons (Schonewille et al., 2010).

We found that anodal stimulation of the cerebellum decreases VOR gain acutely (Figures 4A,B), though we don't see an effect on adaptation-rate like in other studies (Jayaram et al., 2012; Herzfeld et al., 2014; Zuchowski et al., 2014; Avila et al., 2015). We see that VOR gain is reduced prior to the training. Perhaps anodal stimulation induced an acute increase in inhibition by enhancing PC activity. Indeed, others have also reported that artificial activation of PCs may contribute to the induction of VOR gain-down adaptation (Nguyen-Vu et al., 2013). Moreover, a low amplitude external electric field (EEF) is sufficient to modulate PC activity (Chan and Nicholson, 1986; Chan et al., 1988). Together these results suggest that anodal DCS may induce higher PC activity, which in turn could lead to inhibition of its downstream structures.

The possibility that the effects of DCS on plasticity are in part secondary effects on downstream structures comports with there being at least two sites of VOR plasticity (Hansel, 2005): one in the floccular region of cerebellar cortex and one in the VN (Gao et al., 2012). Physiological studies would be necessary to elucidate the relative effects, and these studies would need to include direct measurements from both regions.

We also found that the total gain reduction was similar in the anodal and the cathodal stimulation conditions although the gain reduction at the early phase is clearly different (Figure 4A). In our study, training and testing are assessed post DCS, whereas most of the reports available today are based on stimulation applied during learning. For instance, anodal stimulation facilitates learning in locomotor (Jayaram et al., 2012), force field (Herzfeld et al., 2014), and saccade (Avila et al., 2015) adaptation as well as eye-blink conditioning tasks (Zuchowski et al., 2014), while cathodal stimulation hinders leaning in all these tasks. Surprisingly, the post-stimulation deadaptation curve (Jayaram et al., 2012; Herzfeld et al., 2014) or extinction rate (Zuchowski et al., 2014) shows no difference across various stimulation groups. The later finding is notable because irrespective of altered rate and total amount of learning, polarity has no effect on post-stimulation de-adaptation/ learning processes. In our study, we find that DCS has no post-stimulation effect on the learning phase. Therefore, our study clearly depicts both anodal and cathodal stimulation have short-lasting effects on the habituation phase of the gain-down VOR adaptation task. The de-adaptation experiment (like other studies) is redundant, as we have done all the adaptation training sessions in the post-stimulation period. To discover the actual cause, similar experiments should be performed with a gain increase VOR adaptation paradigm (Gao et al., 2012).

L7-PP2B mice often showed more than one gain during baseline measurements (Figure 6A). Possibly, the eye overshoots the head-position as we have used higher sinusoidal velocity (amplitude of 5° at 1 Hz frequency). We think that sensory signals coming from the parallel fibers fail to excite PC sharply, as there is no LTP in L7-PP2B mice. Therefore, when the high velocity head-movement stops, PCs could not generate sharp inhibition on the VN to stop the eye-movement. A sub-optimal PC inhibition may have caused facilitation of the eye movement in the absence of the head-movement.

We propose three, non-exclusive, possibilities that may explain reduced sensitivity to anodal stimulation in the L7-PP2B mutants: (i) PCs in the mutants may receive more background inhibition; (ii) plasticity at the PC-VN synapses may be essential for VOR gain-down adaptation (De Zeeuw and Ten Brinke, 2015; See CSHP book by Kandel); and/or (iii) plasticity of synapses on PCs in mutants may be saturated, preventing adaptation. The first point reflects the possibility that anodal stimulation may cause inhibition rather than excitation of PCs when there is no LTP or intrinsic plasticity at PCs. Anodal stimulation driven subthreshold depolarization may augment GABA release from molecular layer interneurons (MLI) (Christie et al., 2011; Stagg and Nitsche, 2011) and thereby increase inhibition onto PCs. The second possibility is that anodal DCS has a direct impact on PC-VN plasticity and thereby directly regulates the adaptation process. Loss of PC LTP may retard the effects of anodal stimulation on these synapses. The third reason could be that loss of LTP makes the circuit unresponsive to the pairing of the sensory stimulus with the motor response, as intrinsic plasticity of PCs is also erratic in these mutants (Schonewille et al., 2010). The PP2B transgene may disrupt normal signaling through the PCs or the homeostasis of the network (Lamont and Weber, 2012). This can corrupt the instructive signals sent by PCs to downstream sites like the VN.

Cathodal stimulation induced inhibition of adaptation in L7-PP2B mutants is significantly stronger compared to C57BL/6 mice but similar to the sham group of L7-PP2B mice (Figures 5, 6B). It is evident that this cathodal suppression is a by-product of the mutation of potentiation at the PCs, as these mice fail to learn cerebellar tasks (Schonewille et al., 2010). In addition, we need to examine to what extent long-term depression (LTD) at PF-PC pathway plays a role following cathodal stimulation.

In conclusion, we have successfully developed a mouse model of cerebellar DCS, allowing us to present the first demonstration of cerebellar DCS driven behavioral changes in rodents. We used this model in combination with the popular paradigm of VOR adaptation to test the effect of current stimulation on motor adaptation. The results presented here provide evidence that anodal DCS reduces VOR gain acutely, an effect that is disrupted by ablation of PP2B in PCs. This study, also finds support for recent claims that anodal and cathodal stimulation modulate cerebellar dependent adaptation acutely through distinct pathways. Future research must address the neuronal activity following cerebellar stimulation to understand the spatiotemporal aspects of DCS effects.

## Author contributions

SD, MSp, MF, and OD conceived and designed the experiments. SD performed the experiments. SD and PH analyzed the data. TS helped in building the experimental set-up. SD, OD, MF, MSc, and CD interpreted the data. SD drafted the manuscript. OD, MSc, MF, TS, and PH performed a critical review of the manuscript. CD and MSc were responsible for mutant verification. All the authors read and approved the final version of the manuscript.

### Conflict of interest statement

The authors declare that the research was conducted in the absence of any commercial or financial relationships that could be construed as a potential conflict of interest.
